# Ferroptosis driven by radical oxidation of n-6 polyunsaturated fatty acids mediates acetaminophen-induced acute liver failure

**DOI:** 10.1038/s41419-020-2334-2

**Published:** 2020-02-24

**Authors:** Naoya Yamada, Tadayoshi Karasawa, Hiroaki Kimura, Sachiko Watanabe, Takanori Komada, Ryo Kamata, Ariunaa Sampilvanjil, Junya Ito, Kiyotaka Nakagawa, Hiroshi Kuwata, Shuntaro Hara, Koichi Mizuta, Yasunaru Sakuma, Naohiro Sata, Masafumi Takahashi

**Affiliations:** 1 Division of Inflammation Research, Center for Molecular Medicine, Jichi Medical University, Tochigi, Japan; 2 Division of Gastroenterological, General and Transplant Surgery, Department of Surgery, Jichi Medical University, Tochigi, Japan; 3 Food and Biodynamic Chemistry Laboratory, Graduate School of Agricultural Science, Tohoku University, Sendai, Japan; 4 Division of Health Chemistry, Department of Healthcare and Regulatory Sciences, School of Pharmacy, Showa University, Tokyo, Japan; 5 Department of Transplant Surgery, Saitama Children’s Medical Center, Saitama, Japan

**Keywords:** Physiology, Hepatotoxicity

## Abstract

Acetaminophen (APAP) overdose is a common cause of drug-induced acute liver failure. Although hepatocyte cell death is considered to be the critical event in APAP-induced hepatotoxicity, the underlying mechanism remains unclear. Ferroptosis is a newly discovered type of cell death that is caused by a loss of cellular redox homeostasis. As glutathione (GSH) depletion triggers APAP-induced hepatotoxicity, we investigated the role of ferroptosis in a murine model of APAP-induced acute liver failure. APAP-induced hepatotoxicity (evaluated in terms of ALT, AST, and the histopathological score), lipid peroxidation (4-HNE and MDA), and upregulation of the ferroptosis maker PTGS2 mRNA were markedly prevented by the ferroptosis-specific inhibitor ferrostatin-1 (Fer-1). Fer-1 treatment also completely prevented mortality induced by high-dose APAP. Similarly, APAP-induced hepatotoxicity and lipid peroxidation were prevented by the iron chelator deferoxamine. Using mass spectrometry, we found that lipid peroxides derived from n-6 fatty acids, mainly arachidonic acid, were elevated by APAP, and that auto-oxidation is the predominant mechanism of APAP-derived lipid oxidation. APAP-induced hepatotoxicity was also prevented by genetic inhibition of acyl-CoA synthetase long-chain family member 4 or α-tocopherol supplementation. We found that ferroptosis is responsible for APAP-induced hepatocyte cell death. Our findings provide new insights into the mechanism of APAP-induced hepatotoxicity and suggest that ferroptosis is a potential therapeutic target for APAP-induced acute liver failure.

## Introduction

Acetaminophen (APAP, also known as paracetamol) is one of the most widely used analgesic or antipyretic drugs, and is safe when used at therapeutic doses. However, overdose of APAP causes liver damage in a dose-dependent manner, and in severe cases, results in acute liver failure^1^. Once acute liver failure occurs, liver transplantation is the only established life-saving procedure^2^. Indeed, for decades, APAP-induced hepatotoxicity has been the most common cause of liver transplantation for acute liver failure in western countries^3^. Thus, additional therapeutic approaches for APAP-induced hepatotoxicity are required and research is needed to understand the underlying mechanism. An enormous number of studies on the mechanism underlying APAP-induced hepatotoxicity have been reported so far, and they have clarified the mechanism to some extent^1^. For instance, APAP is metabolized to *N*-acetyl-*p*-benzoquinone imine (NAPQI) in hepatocytes by cytochrome P450 2E1 (CYP2E1)^4^. NAPQI is detoxified by intracellular glutathione (GSH); once the GSH level is decreased, however, highly reactive NAPQI causes hepatotoxicity. Previous investigations have suggested that mitochondrial damage induced by unconjugated NAPQI has a key role in APAP-induced hepatotoxicity^5^. Furthermore, hepatocyte cell death is also considered to be a critical event in APAP-induced hepatotoxicity, and contributes to the development of acute liver failure. However, the molecular mechanism of hepatocyte cell death in this process remains unclear^6–8^.

Iron-dependent cell death (ferroptosis) was originally identified as a new form of regulated cell death in RAS-mutated cancer cells, and occurs when intracellular glutathione peroxidase 4 (GPX4) is inhibited directly or indirectly by a decrease in GSH levels^9,10^. GPX4 inhibition causes the accumulation of iron-dependent lipid peroxide derived from polyunsaturated fatty acid-containing phospholipids, leading to cellular/subcellular membrane damage and eventually cell death without specific effector molecules^9,11,12^. Furthermore, recent studies have demonstrated that ferroptosis has an important role in the pathogenesis of various diseases, including cancer, neurodegenerative diseases, and liver diseases (e.g., hepatic ischemia-reperfusion injury and hemochromatosis)^13,14^. Because intracellular GSH depletion is considered to trigger APAP-induced hepatotoxicity^15^, we hypothesized that ferroptosis is responsible for this disorder. In the present study, using a murine model of APAP-induced acute liver failure, we demonstrated that ferroptosis occurs in the liver after APAP injection and the inhibition of ferroptosis markedly prevents the development of acute liver failure. Furthermore, we explored the molecular mechanism of lipid peroxidation in APAP-induced ferroptosis in vivo. These findings provide new insights into the molecular mechanism underlying APAP-induced acute liver failure and suggest that ferroptosis may be a novel potential therapeutic target for this disorder.

## Materials and methods

### Animal protocols and reagents

All experiments in this study were mainly performed in accordance with the Jichi Medical University Guide for Laboratory Animals (permit No. 17141-01). Wild-type (WT; male, 8–10 weeks old, C57BL6/J background) were purchased from SLC Japan (Shizuoka, Japan). Female acyl-CoA synthetase long-chain family member 4^+/−^ (ACSL4^+/−^) mice were obtained from the European Mouse Mutant Archive, and male ACSL4^−/Y^ were generated by interbreeding male WT (ACSL4^+/Y^) mice with female ACSL4^+/−^ mice. The experiments using ACSL4^−/Y^ mice and their ACSL4^+/Y^ littermates (8–10 weeks old, C57BL6/J background) were performed at Showa University (Tokyo, Japan) in accordance with the Institutional Guide for Laboratory Animals (permit No. 29026). Mice were housed in an environment maintained at 23 ± 2 °C with ad libitum access to food and water under a 12-h light/dark cycle with lights on from 8:00 to 20:00. APAP was purchased from Sigma-Aldrich (St. Louis, MO) and dissolved in 37 °C warmed saline. Ferrostatin-1 (Fer-1) and α-tocopherol (α-Toc) were purchased from Cayman Chemical (Ann Arbor, MI) and Wako Chemicals (Osaka, Japan), respectively, and dissolved in PEG400 + TWEEN 8+ DW. Deferoxamine (DFO) was purchased from Abcam (Cambridge. MA) and dissolved in PBS. Other reagents were obtained from Sigma-Aldrich unless otherwise specified.

To establish a murine model of APAP-induced acute liver failure, overnight-fasted mice (17:00–9:00, 16 h) were injected intraperitoneally with APAP (100–400 mg/kg) or vehicle (control). Fasting was maintained for 6 h after APAP injection. Fer-1 (10 mg/kg) and α-Toc (100 mg/kg) were intraperitoneally injected in mice 1 h prior to APAP injection. DFO (100 mg/kg/day) was intraperitoneally injected for 7 consecutive days prior to APAP injection. Mice were randomly divided into different groups (no blinding was done).

### Measurement of serum parameters and iron content

Serum levels of aspartic aminotransferase (AST) and alanine aminotransferase (ALT) were measured using a Fuji-DRYCHEM analyzer (Fuji Film, Tokyo, Japan) according to the manufacturer’s instructions. Liver tissue samples were homogenized in saline, and iron (Fe) concentrations were measured by Nitroso-PSAP using a Hitachi 7180 (Hitachi, Tokyo, Japan).

### Histology and immunohistochemistry

Histology and immunohistochemistry were performed as described previously^16^. Paraffin-embedded tissue sections were stained with hematoxylin and eosin. The severity of liver injury was graded according to the histopathology score commonly used for APAP hepatotoxicity on a scale from 0 to 5^17^. Immunohistochemical analysis was performed using the following Abs: the lipid peroxidation marker 4-hydroxyl-2-noneal (4-HNE; clone HNEJ-2, Japan Institute for the Control of Aging, Nikken SEIL, Shizuoka, Japan), acyl-CoA synthetase long-chain family member 4 (ACSL4; ab155282, Abcam, Cambridge, MA), and the pan-leukocyte marker CD45 (#550539, BD Biosciences, Franklin Lakes, NJ). Terminal deoxynucleotidyl transferase dUTP nick end labeling (TUNEL) stain was performed using MEBASTAIN Apoptosis TUNEL Kit Direct (MBL, Aichi, Japan) according to the manufacturer’s instructions. The stained sections were analyzed using a microscope (FSX-100; Olympus, Tokyo, Japan).

### Real-time reverse transcription-polymerase chain reaction (RT-PCR)

Total RNA was prepared using ISOGEN (Nippon Gene Co., Ltd., Toyama, Japan) according to the manufacturer’s instructions. Real-time RT-PCR analysis was performed using the Thermal Cycler Dice Real-Time System II (Takara Bio Inc., Shiga, Japan) to detect the mRNA expression of CYP2E1, PTGS2, IL-1β, IL-6, TNF-α, CCL2, Ly6G, EMR1, and β-actin. The primers are listed in Supplementary Table 1. The expression levels of each target gene were normalized by subtracting the corresponding β-actin threshold cycle (CT) value; normalization was carried out using the ΔΔCT comparative method.

### Western blotting

Western blotting was performed as described previously^18^ using primary antibodies for xCT (#26864-1-AP, Proteintech, Tokyo, Japan), GPX4 (sc-166570, Santa Cruz Biotechnology, Dallas, TX), CYP2E1 (#19937-1-AP, Proteintech, Tokyo, Japan), caspase-3 and cleaved caspase-3 (#9665, Cell Signaling Technology, Inc., Boston, MA), and receptor-interacting protein kinase 3 (RIPK3; #95702, Cell Signaling Technology, Inc., Boston, MA). Immunoreactive bands were visualized by Western BLoT Quant HRP Substrate (Takara Bio Inc.). The expression levels of β-actin served as an internal control for protein loading.

### Measurement of MDA and GSH

Malondialdehyde (MDA) was evaluated by a TBARS Assay Kit (Cayman Chemical, Ann Arbor, MI) according to the manufacturer’s instructions. Total GSH and glutathione disulfide (oxidized glutathione, GSSG) were evaluated by a GSSG/GSH Quantification Kit (Dojindo, Kumamoto, Japan) according to the manufacturer’s instructions. The amount of reduced GSH (shown as GSH) was calculated as total GSH−2GSSG.

### LC-MS analysis for lipid mediators

Lipid mediators in the liver tissue were analyzed by an LC-MS-8060 (Shimadzu, Kyoto, Japan) as described previously^19^. The detailed methods are described in the Supplementary Information.

### Analysis of lipid hydroperoxide isomers

Lipid hydroperoxide isomers in the serum were analyzed by using LC-MS/MS as described previously^20,21^. The detailed methods are described in the Supplementary Information.

### Hydrodynamics-based transfection using a CRISPR/Cas9 system in vivo

sgRNAs targeting the ACSL4 and green fluorescent protein (GFP) genes were designed by CRISPR direct (https://crispr.dbcls.jp/)^22^. DNA sequences expressing sgRNAs of ACSL4 and GFP genes were cloned into Bbs I-linearized pX330 vector (Addgene, #42230). The DNA sequences were as follows: px330-ACSL4-FP, CACCGAAGTGTGTGACAGAGCGATA; px330-ACSL4-RP, AAACTSTCGCTCTGTCACACACTTC; px330-GFP-FP, CACCGGAGCTGGACGGCGACGTAAA px330-GFP-RP, AAACTTTACGTCGCCGTCCAGCTCC. Under general anesthesia with isoflurane, mice were rapidly injected with 10 µg (diluted in 1.6 mL saline) of px330-sgACSL4 or px330-sgGFP via the penile vein, and 7 days later injected with APAP (Supplementary Fig. S1).

### Statistics

Data are expressed as the mean ± SEM. Data were analyzed by Student’s *t* test or the Mann–Whitney test to evaluate the differences between two groups. For comparisons between multiple groups, the significance of differences between group means was determined by the Kruskal–Wallis test or Mann–Whitney test with the Bonferroni correction. All analyses were performed using GraphPad Prism version 7 (San Diego, CA). A *p* value of < 0.05 was considered to be statistically significant.

## Results

### Ferroptosis mediates APAP-induced hepatotoxicity and mortality

**Fig. 1:**
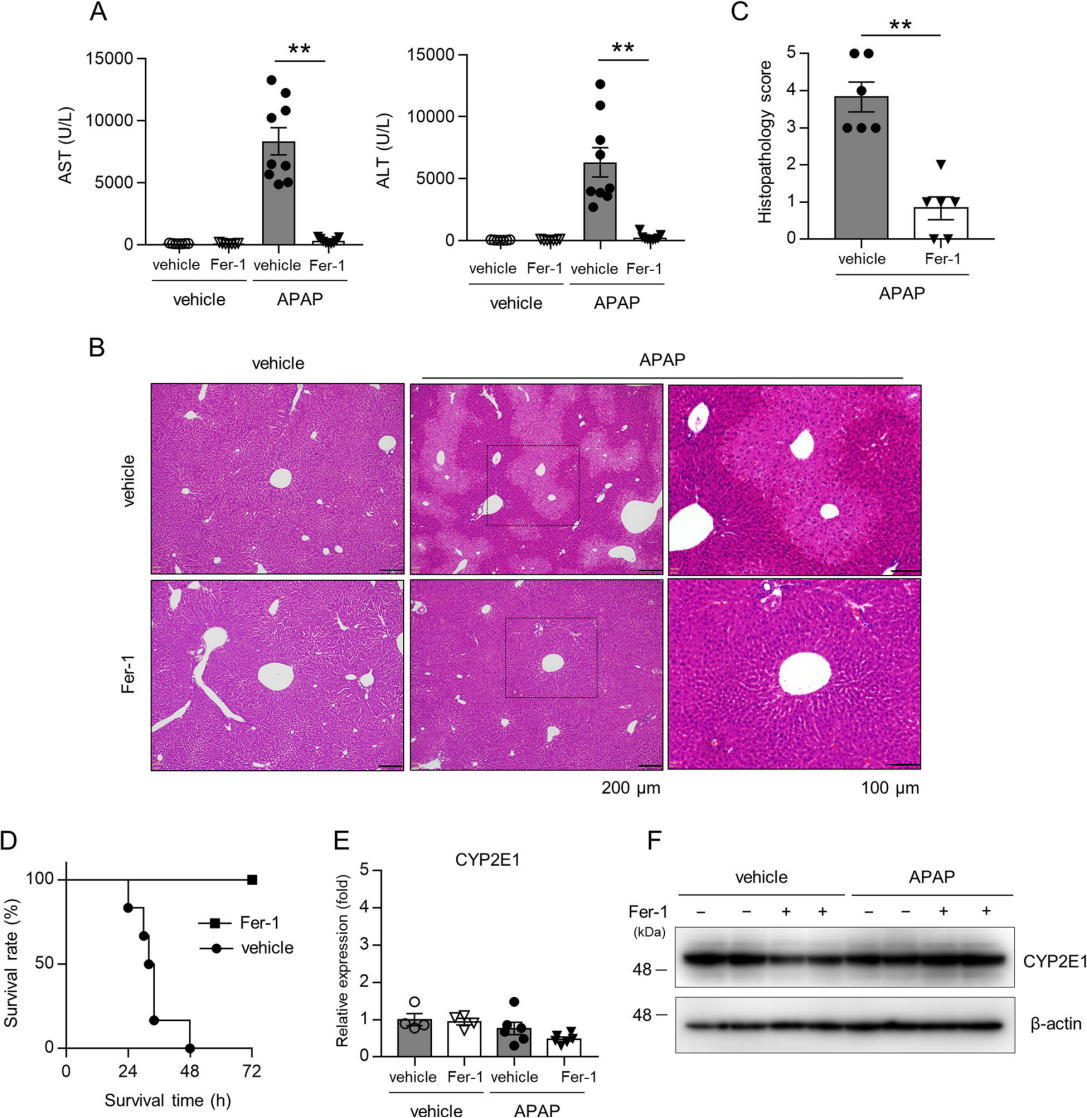
Ferroptosis mediates APAP-induced hepatotoxicity and lethality. Liver and serum samples were obtained from mice injected with vehicle or APAP (200 mg/kg) 3 h after injection. Mice treated with Fer-1 (10 mg/kg) or vehicle 1 h prior to injection. **a** Serum levels of AST and ALT were assessed (*n* = 7–9 for each). **b**, **c** Liver sections were stained with HE, and histopathology scores were assessed (*n* = 4–6 for each). **d** Survival time after injection of a lethal dose of APAP (400 mg/kg) was analyzed using the Kaplan–Meier method (*n* = 4–6 each). **e** Hepatic CYP2E1 mRNA expression was assessed (*n* = 4–6 for each). **f** Liver samples were analyzed by western blotting for CYP2E1. β-actin served as a loading control. Statistical significance was calculated using the Mann–Whitney test **c**–**e** with the Bonferroni correction. Data are expressed as dot plots and/or means ± SEM. *p < 0.05, **p < 0.01.

To investigate the role of ferroptosis in APAP-induced hepatotoxicity, we examined the effect of Fer-1, a specific inhibitor of ferroptosis^10^, on liver damage in WT mice injected with APAP. Serum levels of ALT and AST were clearly elevated at 3 h after APAP (200 mg/kg) injection, and the elevated levels of ALT and AST were significantly lower in mice treated with Fer-1 (10 mg/kg) 1 h prior to APAP injection (Fig. 1a). The inhibitory effects of Fer-1 on the APAP-induced elevation of serum ALT and AST were dose- and time-dependent (100–200 mg/kg, 3–24 h) (Supplementary Fig. S2). Consistent with finding, the histological analysis showed that severe centrilobular necrotic change (acinar zone 3), which is characteristic of APAP-induced hepatotoxicity^17^, was observed in the liver at 3 h after APAP (200 mg/kg) injection, and this necrotic change was dramatically improved in mice treated with Fer-1 (Fig. 1b). Histopathological severity (hepatotoxicity score) was also markedly improved by Fer-1 treatment (Fig. 1c). To further investigate the role of ferroptosis, we monitored mice over 72 h after the injection of a high dose of APAP (400 mg/kg) and found that, while all mice treated with vehicle died within 48 h, none of those treated with Fer-1 died (Fig. 1d). To rule out the possibility that Fer-1 could change APAP metabolism, we assessed the expression of CYP2E1, a key enzyme to metabolize APAP to NAPQI, and showed that Fer-1 had no effect on hepatic expression of CYP2E1 mRNA and protein (Fig. 1e, f).

### APAP induces PTGS2 expression, lipid peroxidation, and GSH depletion

**Fig. 2:**
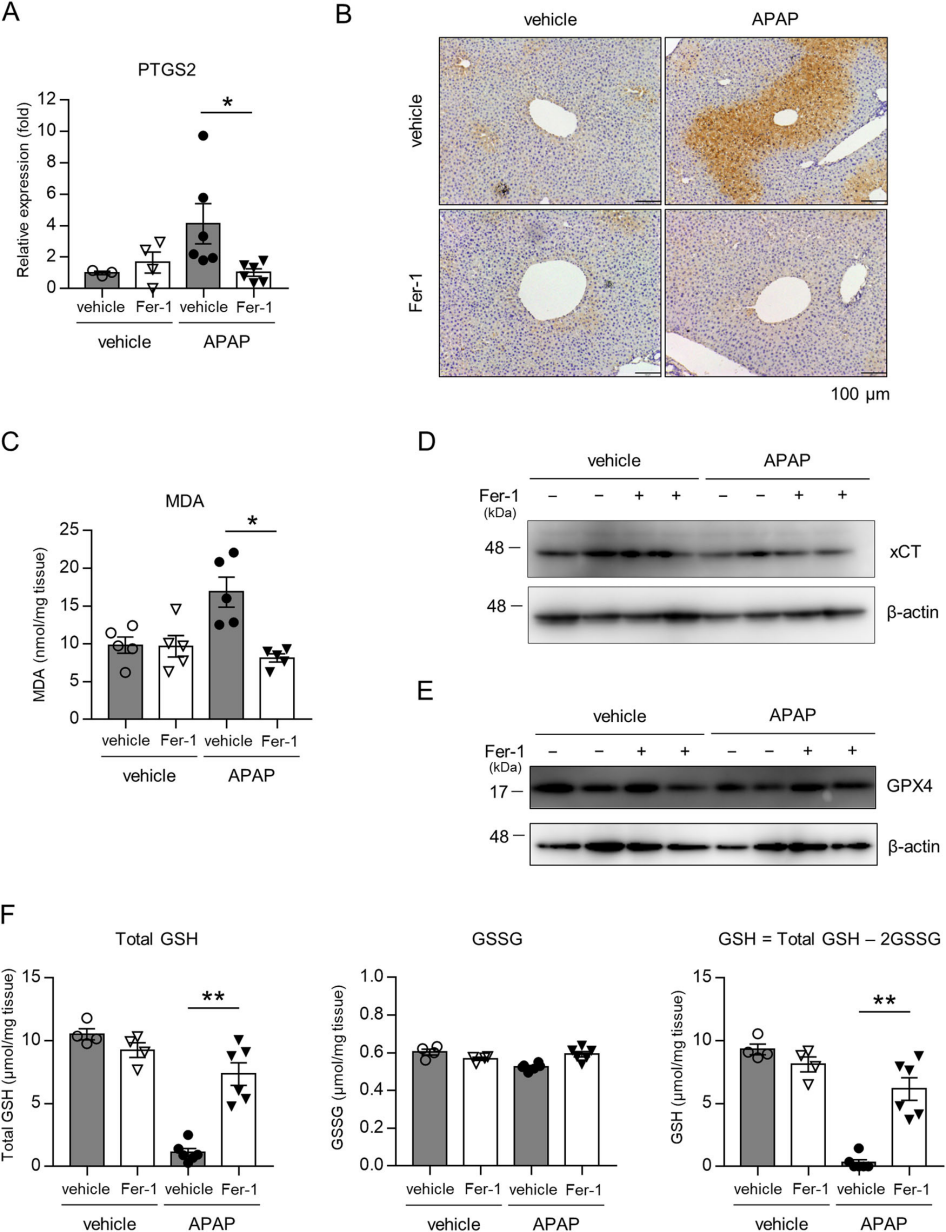
APAP induces Ptgs2 expression, lipid peroxidation, and GSH depletion. Liver samples were obtained from mice injected with vehicle or APAP (200 mg/kg) 3 h after injection. Mice were treated with Fer-1 (10 mg/kg) or vehicle 1 h prior to injection. **a** Hepatic PTGS2 mRNA expression was assessed (*n* = 4–6 for each). **b** Liver sections were analyzed by immunohistochemical staining for 4-HNE (*n* = 4–6 for each). **c** Hepatic MDA levels were assessed by MDA-TBARS assay (*n* = 5 for each). **d** Liver samples were analyzed by western blotting for xCT. β-actin served as a loading control. **e** Liver samples were analyzed by western blotting for GPX4. β-actin served as a loading control. **f** Hepatic levels of total GSH (GSH + 2GSSG) and reductant GSH were assessed. GSH was calculated as total GSH –2GSSG (*n* = 4–6 for each). Statistical significance was calculated using the Mann–Whitney test with the Bonferroni correction **a**–**e**. Data are expressed as dot plots and/or means ± SEM. *p < 0.05, **p < 0.01.

We next assessed key features of ferroptosis including mRNA expression of PTGS2 (encoding COX-2) and lipid peroxidation markers^12^. Real-time RT-PCR analysis showed that PTGS2 mRNA levels were increased in the liver of APAP-injected mice, and this increase was significantly reduced by Fer-1 (Fig. 2a). In addition, the expression of 4-HNE, a secondary product of lipid peroxidation, was clearly visualized in the zone 3 area damaged by APAP, but was not detected in mice treated with Fer-1 (Fig. 2b). As the excessive accumulation of 4-HNE has also been shown to promote other types of cell death, such as apoptosis and necrosis^23^, we measured MDA, another secondary product of lipid peroxidation. A similar pattern of hepatic MDA levels was observed (Fig. 2c). As ferroptosis occurs when GPX4 is inhibited either directly through its expression level or indirectly through the unavailability of GSH^9^, we performed western blotting for xCT and GPX4 and showed that the xCT and GPX4 levels in the liver did not differ between vehicle and APAP-injected mice (Fig. 2d, e). On the other hand, the hepatic levels of total GSH (GSH+2GSSG) and reductant GSH were clearly decreased by APAP injection, and this GSH depletion was significantly restored by Fer-1, whereas there was no change in GSSG levels (Fig. 2f).

### Fer-1 has no effects on other types of cell death or inflammatory responses

**Fig. 3:**
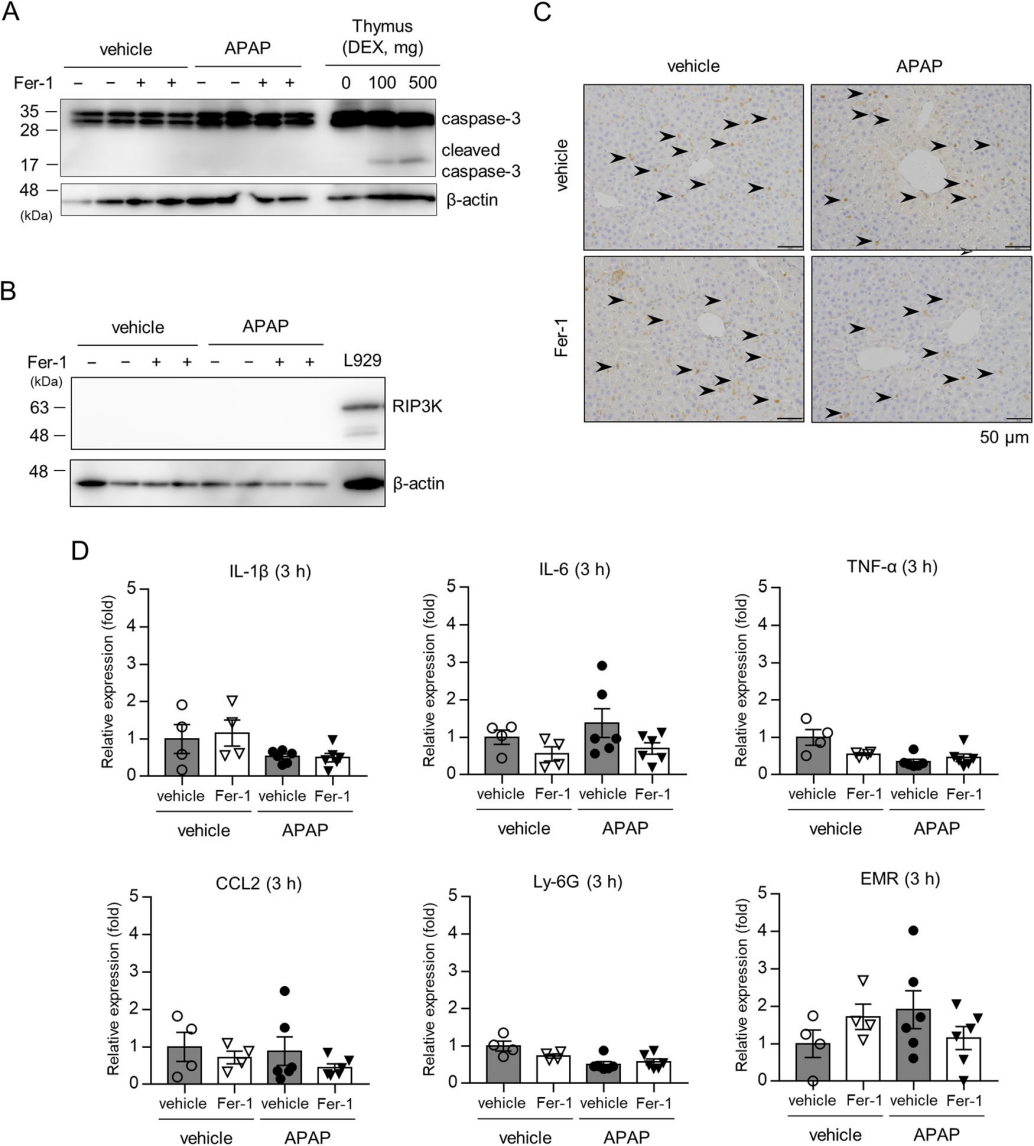
Fer-1 has no effects on other types of cell death or inflammatory responses. Liver samples were obtained from mice injected with vehicle or APAP (200 mg/kg) 3 h after injection. Mice were treated with Fer-1 (10 mg/kg) or vehicle 1 h prior to injection. **a**, **b** Liver samples were analyzed by western blotting for caspase-3, cleaved caspase-3, and RIP3K. DEX-treated thymus and L929 cells were used as positive controls for cleaved caspase-3 and RIP3K, respectively. β-actin served as a loading control. **c** Liver sections were analyzed by immunohistochemical staining for CD45 (*n* = 4–6 for each). **d** Hepatic mRNA expression of inflammatory cell markers (Ly6G and EMR) and cytokines (IL-1β, IL-6, TNF-α, and CCL2) was assessed (*n* = 4–6 for each). Statistical significance was calculated using the Mann–Whitney test with the Bonferroni correction **a**–**d**. Data are expressed as dot plots and/or means ± SEM.

To rule out the possibility that other types of cell death, such as apoptosis and necroptosis, are involved in APAP-induced hepatotoxicity, we assessed the expression of caspase-3, cleaved caspase-3, and RIP3K to analyze apoptosis and necroptosis, respectively. Western blot analysis showed no expression of cleaved caspase-3 or RIP3K in the liver of APAP-injected mice treated with vehicle or Fer-1 (Fig. 3a). Consistent with this finding, no TUNEL-positive cells were detected in the liver (Supplementary Fig. S3). As inflammation is associated with APAP-induced hepatotoxicity^24^, we next performed an immunohistochemical analysis for the pan-leukocyte marker CD45. Contrary to our expectation, there was no difference in inflammatory cell infiltration in the liver at the early phase (3 h) among vehicle- and APAP-injected mice pretreated with or without Fer-1 (Fig. 3c–e). Consistent with this finding, real-time RT-PCR analysis showed no significant differences in the expression of inflammatory cytokines (IL-1β, IL-6, TNF-α, and CCL2) or cell markers (Ly6G for neutrophils and EMR1 for macrophages) in the liver among these mice (Fig. 3d).

### Iron chelator inhibits APAP-induced hepatotoxicity and lipid peroxidation

**Fig. 4:**
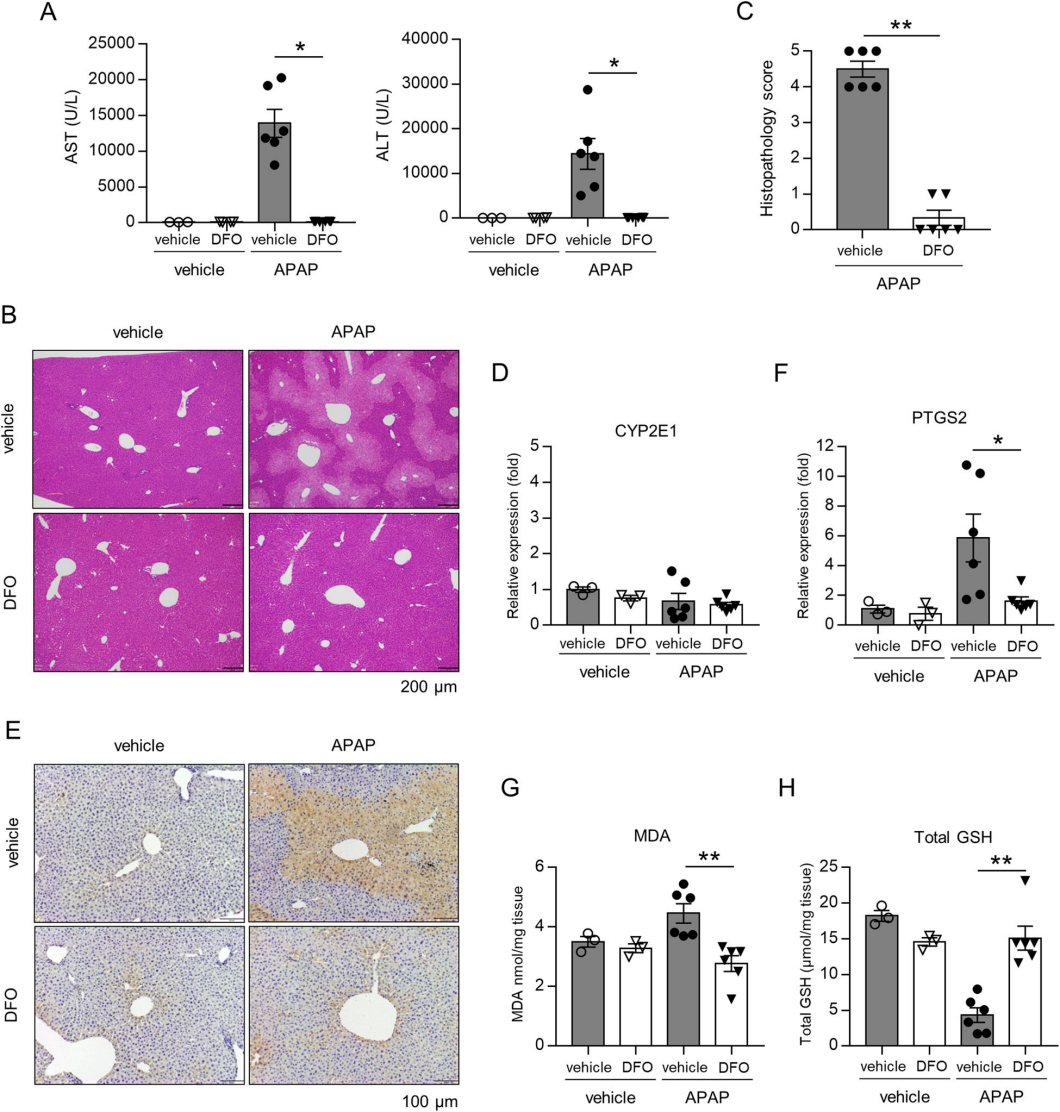
Iron chelator inhibits APAP-induced hepatotoxicity and lipid peroxidation. Liver and serum samples were obtained from mice injected with vehicle or APAP (200 mg/kg) 3 h after injection. Mice were treated with DFO (100 mg/kg/day) or vehicle for 7 consecutive days prior to APAP injection. **a** Serum AST and ALT levels. **b**, **c** HE staining and histopathology score. **d** Hepatic CYP2E1 mRNA expression. **e** Immunostaining for 4-HNE. **f** Hepatic PTGS2 mRNA expression. **g** Hepatic MDA levels. **h** Hepatic GSH levels. Statistical significance was calculated using the Mann–Whitney test **c**–**e** with the Bonferroni correction. Data are expressed as dot plots and/or means ± SEM (*n* = 6–8 for each). *p < 0.05, **p < 0.01.

Ferroptosis is an iron-dependent and peroxidation-driven form of cell death that is inhibited by the chelation of iron;^10^ therefore, we next administrated the iron chelator DFO (100 mg/kg/day) to mice for 7 consecutive days prior to APAP injection. DFO treatment clearly inhibited APAP-induced hepatotoxicity, as determined by serum ALT and AST elevation and zone 3 necrotic changes in the liver (Fig. 4a–c). Similar to Fer-1 treatment, DFO had no effect on hepatic CYP2E1 expression (Fig. 4d), but inhibited PTGS2 mRNA expression and lipid peroxidation determined by the expression of 4-HNE and the levels of MDA and total GSH (Fig. 4e–h). Reduction of iron content by DFO treatment was confirmed in liver tissues (Supplementary Fig. S4).

### Arachidonic acid-derived lipid peroxidation is involved in APAP-induced hepatotoxicity

**Fig. 5:**
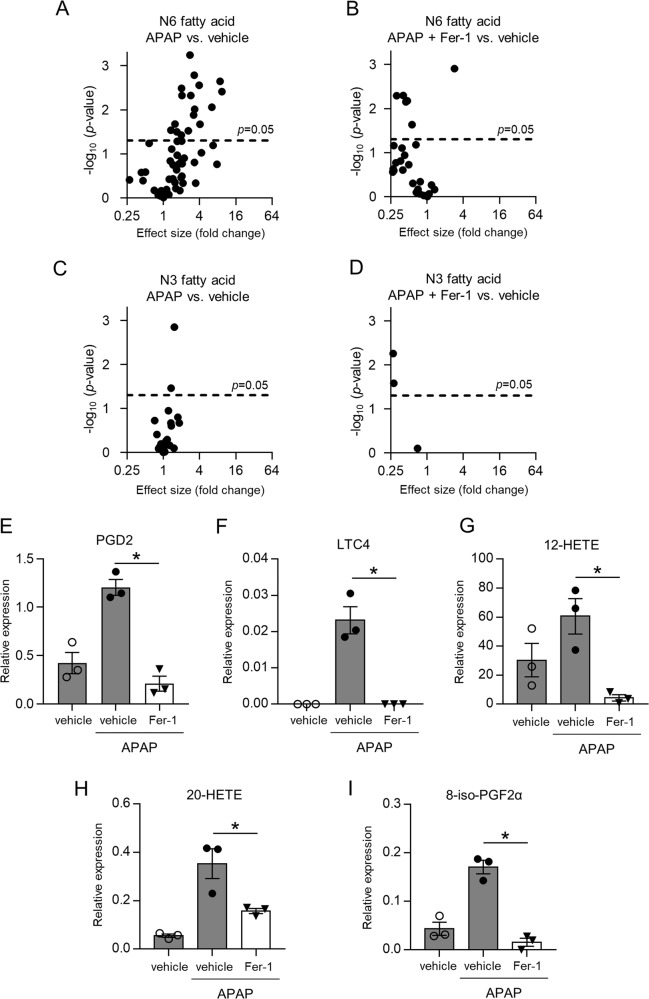
Arachidonic acid-derived lipid peroxidation is involved in APAP-induced hepatotoxicity. Liver samples were obtained from mice injected with vehicle or APAP (200 mg/kg) 3 h after injection. Mice were treated with Fer-1 (10 mg/kg) or vehicle 1 h prior to injection. **a**–**d** Liver samples were analyzed by LC-MS to identify lipid mediators and volcano plots are shown. **a** APAP vs. vehicle in n-6 fatty acid, **b** APAP + Fer-1 vs. vehicle in n-6 fatty acid, **c** APAP vs. vehicle in n-3 fatty acid, **d** APAP + Fer-1 vs. vehicle in n-3 fatty acid. **e**–**h** Relative expression levels of PGD2, LTC4, 12-HETE, 20-HETE, and 8-iso-PGF2α are shown. The data are expressed as relative expression compared with a reference standard (*n* = 3 for each). Statistical significance was calculated using Student’s *t* test **e**–**h**. Data are expressed as dot plots and/or means ± SEM. *p < 0.05, **p < 0.01.

To explore the molecular mechanism by which APAP induces lipid peroxidation and subsequent ferroptosis in the liver, we measured the concentrations of 158 lipid mediators in the liver tissue 3 h after APAP injection by using LC-MS, and found that lipid peroxides derived from n-6 polyunsaturated fatty acid (PUFA), including arachidonic acid (AA), linoleic acid (LA), and dihomo-γ-linolenic acid (DGLA), were elevated by APAP injection, and the elevation of these lipid peroxides was inhibited by Fer-1 (Fig. 5a, b, Supplementary Excel file). In contrast, there was no significant change in lipid peroxides derived from n-3 fatty acid between vehicle- and APAP-injected mice (Fig. 5c, d). Arachidonic acids are known to be modified by cyclooxygenase, lipoxygenase, cytochrome P450 pathways, and/or auto-oxidation (radical oxidation)^23,25^. Indeed, the levels of prostaglandin D2 (cyclooxygenase pathway), leukotriene C4 and 12-hydroxyeicosatetraenoic acid (12-HETE, lipoxygenase pathway), and 20- hydroxyeicosatetraenoic acid (HETE; cytochrome P450 pathway), and 8-iso-Prostaglandin F2α (PGF2α; auto-oxidation) were elevated in APAP-injected mice, and this elevation was inhibited by Fer-1 (Fig. 5e–h), suggesting that non-specific pathways of arachidonic acid metabolism are involved in APAP-induced hepatotoxicity. In particular, as the recent report suggests, the elevation of 8-iso-PGF2α levels suggests that auto-oxidation may be the predominant mechanism of APAP-induced lipid peroxidation^26^.

### ACSL4 deletion attenuates APAP-induced hepatotoxicity and lipid peroxidation

**Fig. 6:**
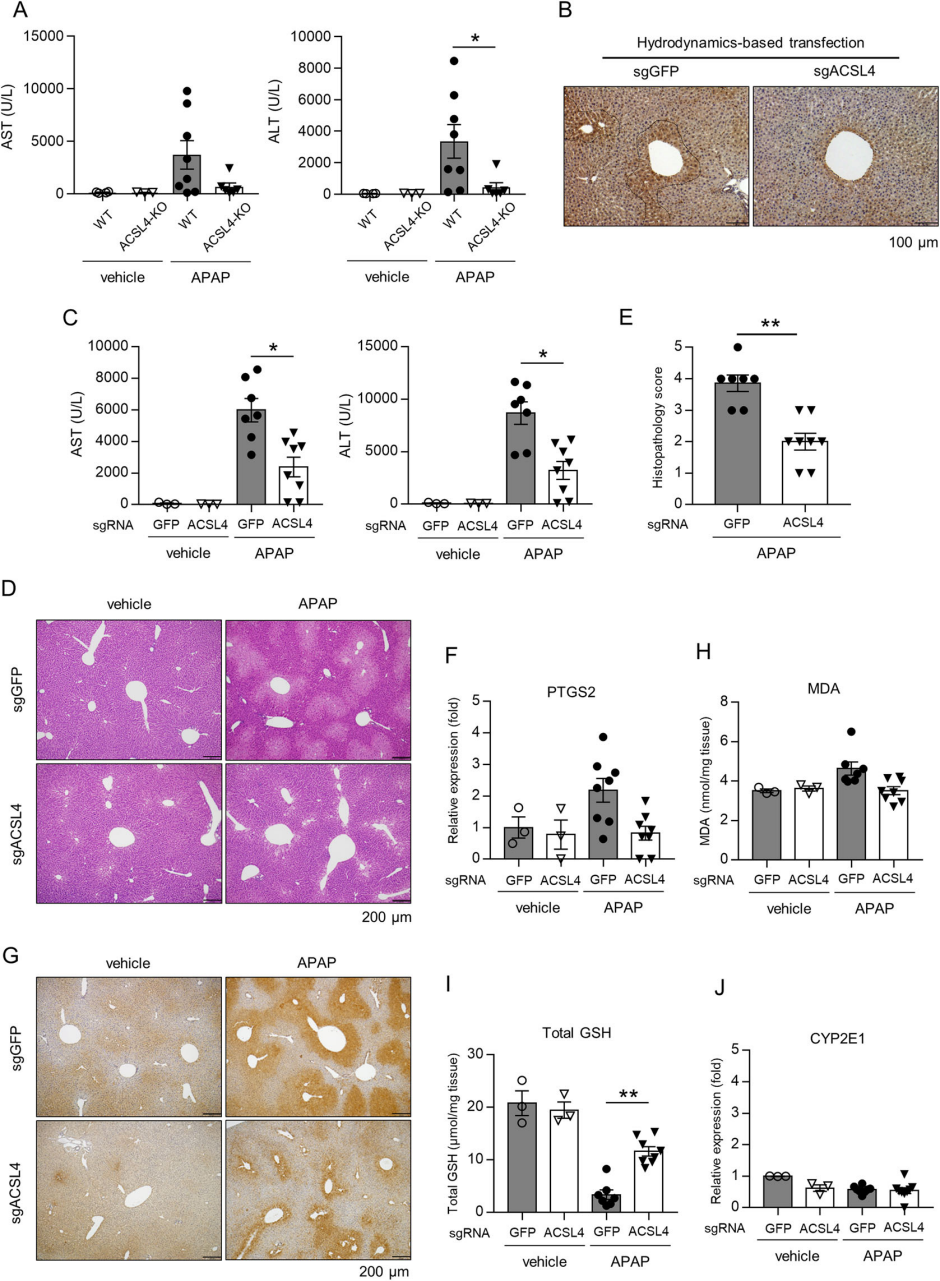
ACSL4 deletion attenuates APAP-induced hepatotoxicity and lipid peroxidation. **a** Liver and serum samples were obtained from ACSL4^+^^/Y^ and ACSL4^–/Y^ mice injected with vehicle or APAP (200 mg/kg) 3 h after injection. Serum levels of AST and ALT were assessed (*n* = 3–5 for each). **b**–**e** The hydrodynamics-based transfection method of CRISPR/Cas9 genome editing was used to create hepatic ACSL4-deleted mice. Liver and serum samples were obtained from mice injected with vehicle or APAP (200 mg/kg) 3 h after injection. px330-ACSL4-sgRNA or GFP-sgRNA was injected by the hydrodynamics-based method 7 days prior to APAP injection. **b** Hepatic ACSL4 expression. **c** Serum AST and ALT levels (*n* = 3–5 for each). **d** HE staining. **e** Histopathological score. **f** Hepatic PTGS2 mRNA expression. **g** 4-HNE immunostaining. **h** Hepatic MDA levels. **i** Hepatic total GSH levels. **j** Hepatic CYP2E1 mRNA expression. Statistical significance was calculated using the Mann–Whitney test with the Bonferroni correction **a**–**e**. Data are expressed as dot plots and/or means ± SEM. *p < 0.05, **p < 0.01 (*n* = 6–8 for each).

As ACSL4 is considered to be a key enzyme to trigger ferroptosis^9,11,27^, we next examined the role of ACSL4 in APAP-induced hepatotoxicity by two strategies: ACSL4^–/Y^ mice and CRISPR/Cas9-mediated ACSL4 deletion in the liver. The elevation of serum ALT and AST by APAP was significantly attenuated in ACSL4^–/Y^ mice, compared with that in their ACSL4^+^^/Y^ littermates (Fig. 6a). To investigate the specific role of ACSL4 in hepatocytes, we used the hydrodynamics-based transfection method of CRISPR/Cas9 genome editing in mice, which has been reported to be an effective strategy for gene deletion in the liver^28^. Fatty-acid metabolism by enzymes including ACSL4 generally occurs in the zone 3 area of the liver^29^. Indeed, we observed that ACSL4, 4-HNE, and CYP2E1 were expressed in the zone 3 area in the liver (Supplementary Fig. S5). We showed that intravenous injection of CRISPR/Cas9 vector, px330-ACSL4-sgRNA, apparently reduced ACSL4 expression in the zone 3 area (Fig. 6b). The transfection of px330-ACSL4-sgRNA attenuated liver damage (evaluated in terms of serum ALT and AST levels, and the histopathology score), lipid peroxidation (4-HNE, MDA, and total GSH), and PTGS2 mRNA expression, compared with transfection with px330-GFP-sgRNA (Fig. 6c–i). Similar to the data with Fer-1 and DFO, the transfection of px330-ACSL4-sgRNA had no effect on CYP2E mRNA expression (Fig. 6j).

### Mechanisms of lipid peroxidation in APAP-induced hepatotoxicity

**Fig. 7:**
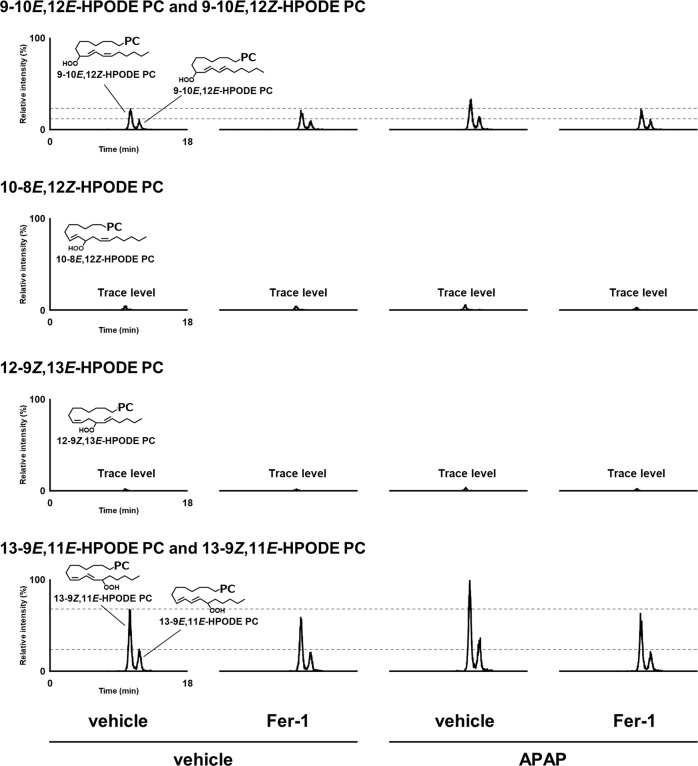
Evaluation of lipid peroxidation mechanisms via analysis of lipid hydroperoxide isomers. MRM chromatograms of PCOOH isomers in serum. Serum samples were obtained from mice injected with vehicle or APAP (200 mg/kg) 3 h after injection. Mice were treated with Fer-1 (10 mg/kg) or vehicle 1 h prior to injection. Extract from a serum sample (10 µL) was analyzed by HPLC-MS-MS. Detailed analytical conditions are described in the Supplementary Information.

Lipid peroxidation is caused by three oxidation mechanisms: auto-oxidation, enzymatic oxidation, and singlet oxygen-induced oxidation^30,31^. To evaluate the mechanisms of lipid peroxidation by APAP injection, we assessed six isomers of phosphatidylcholine hydroperoxide (PCOOH; 9-10*E*,12*Z*-HPODE PC, 9-10*E*,12*E*-HPODE PC, 10-8*E*,12*Z*-HPODE PC, 12-9*Z*,13*E*-HPODE PC, 13-9*Z*,11*E*-HPODE PC, and 13-9*E*,11*E*-HPODE PC) by using LC-MS/MS. PCOOH isomers were detected in serum samples collected 3 h after APAP injection (Fig. 7). Of these, the isomers that were characteristic of auto-oxidation (i.e., 9-10*E*,12*Z*-HPODE PC, 9-10*E*,12*E*-HPODE PC, 13-9*Z*,11*E*-HPODE PC, and 13-9*E*,11*E*-HPODE PC) were clearly detected, whereas those caused by singlet oxygen-induced oxidation (i.e., 10-8*E*,12*Z*-HPODE PC and 12-9*Z*,13*E*-HPODE PC) were only detected at trace levels. Moreover, serum levels of auto-oxidation-driven PCOOH isomers were increased by APAP, and this increase was suppressed by Fer-1 (Supplementary Fig. S6). These results suggest that auto-oxidation is the predominant mechanism to induce lipid oxidation in APAP-induced hepatotoxicity.

### α-Toc prevents APAP-induced hepatotoxicity and lipid peroxidation

**Fig. 8:**
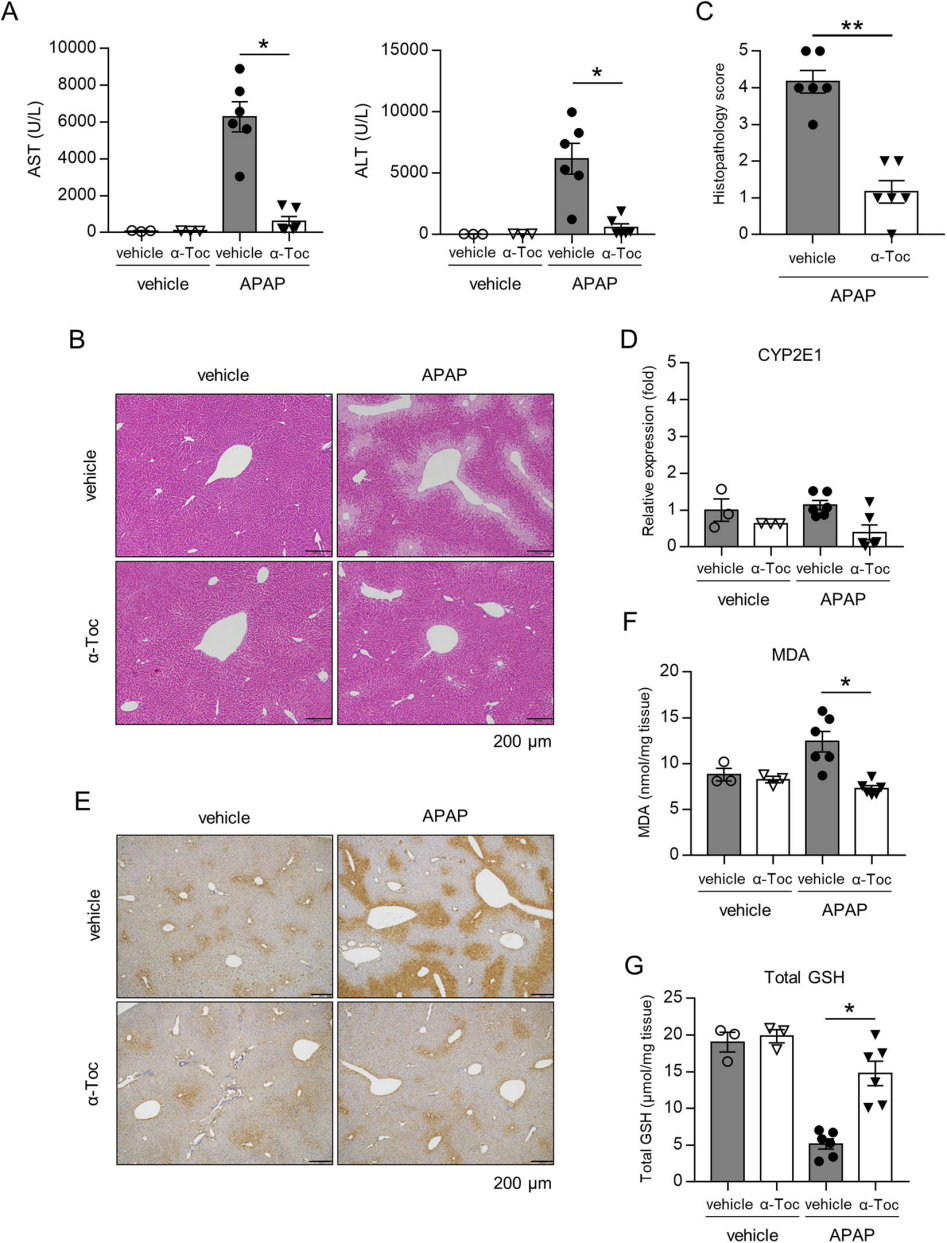
α-Tocopherol inhibits APAP-induced hepatotoxicity and lipid peroxidation. Liver and serum samples were obtained from mice injected with vehicle or APAP (200 mg/kg) 3 h after injection. Mice were treated with α-tocopherol (α-Toc, 100 mg/kg/day) or vehicle 1 h prior to injection. **a** Serum AST and ALT levels. **b**, **c** HE staining and histopathology score. **d** Hepatic CYP2E1 mRNA expression. **e** 4-HNE immunostaining. **f** Hepatic MDA levels. **g** Hepatic total GSH levels. Statistical significance was calculated using Mann–Whitney test **a**–**g** with the Bonferroni correction. Data are expressed as dot plots and/or means ± SEM (*n* = 3–6 for each). *p < 0.05, **p < 0.01.

To explore a potential clinical application, we finally tested the effect of α-Toc (vitamin E) on APAP-induced hepatotoxicity because it has been used as a common lipophilic antioxidant in clinical practice^32^ and has been shown to exert a radical trapping effect similar to Fer-1^33^. As expected, supplementation with α-Toc clearly attenuated liver damage (evaluated in terms of serum ALT and AST levels, and the histopathology score) and lipid peroxidation (4-HNE, MDA, and total GSH), but had no effect on CYP2E1 mRNA expression (Fig. 8).

## Discussion

The major findings of this study are as follows: (1) APAP-induced hepatotoxicity, lipid peroxidation, and upregulation of the ferroptosis maker PTGS2 were markedly prevented by the ferroptosis-specific inhibitor Fer-1. Furthermore, high-dose APAP-induced lethality was completely prevented by Fer-1; (2) APAP failed to induce the hepatic expression of cleaved caspase-3, RIPK3, and inflammatory markers at the early phase after APAP injection; (3) APAP-induced hepatotoxicity and lipid peroxidation were completely prevented by DFO; 4 Mass spectrometry revealed that lipid peroxides derived from n-6 PUFA were elevated by APAP, and that auto-oxidation is the predominant mechanism of APAP-induced lipid oxidation; and (5) APAP-induced hepatotoxicity was prevented by genetic ACSL4 inhibition or α-Toc supplementation. In the present study, we found that ferroptosis, a newly discovered type of cell death, is the initial and predominant event during APAP-induced hepatotoxicity. We also found that APAP-induced hepatic lipid peroxidation of n-6 PUFA mainly through auto-oxidation. These findings clarify the molecular events underlying APAP-induced hepatotoxicity and suggest that ferroptosis is a novel therapeutic target for APAP-induced acute liver failure.

APAP-induced acute liver failure is a common cause of acute liver failure and has become a major medical problem in western countries^34^. APAP-induced hepatotoxicity is mainly caused by the highly reactive metabolite NAPQI derived from APAP^5^. APAP-derived NAPQI is generally conjugated with sulfhydryl groups of GSH for detoxification; however, APAP overdose causes the excessive formation of NAPQI, which depletes GSH stores and binds covalently to cellular proteins, thereby leading to oxidative injury and cell death in hepatocytes. It is worth noting that GSH depletion by APAP administration was rescued by Fer-1, DFO, and genetically ACSL4 deletion, which suggests that GSH depletion in APAP hepatotoxicity is triggered by not only NAPQI-GSH adducts, but also lipid peroxides (i.e., LOOH + 2GSH → LOH + H_2_O + GSSG). Furthermore, lipid peroxides reacts with metals including iron and changes to alkylperoxyl radical, resulting in many reactive carbonyls (e.g., 4-HNE and MDA). These reactive carbonyls are strongly conjugated to GSH and promotes intracellular GSH depletion. Thus, we speculate that initial GSH depletion is triggered by NAPQI-GSH adducts formation, but multistage reaction is involved in subsequent GSH depletion.

Previous studies have suggested that several types of regulated cell death, including apoptosis and necroptosis, are involved in APAP-induced hepatotoxicity^6–8^. It is currently accepted that apoptosis has a very limited role in this process owing to the lack of efficacy of pan-caspase inhibitors in rodent models of APAP-induced hepatotoxicity^6^. In addition, because the morphological features of APAP-induced cell death include cell swelling, karyolysis, release of cell contents, and inflammation, necroptosis has received considerable attention. Necroptosis is characterized by receptor-interacting protein kinase (RIPK) and its substrate mixed-lineage kinase domain-like (MLKL) activation, both of which are biomarkers for assessing necroptosis^35^. Although the RIPK1 inhibitor necrostatin-1 (Nec-1) has previously been reported to have a protective effect in APAP-induced acute liver failure^36^, Nec-1 also has off target effects, and therefore, the exact contribution of necroptosis in mice genetically modified for RIPK and MLKL still needed to be evaluated. Recently, several groups have failed to show that it protects against APAP-induced acute liver failure in mice deficient in RIPK3 and MLKL;^8^ therefore, it is now accepted that necroptosis is not responsible for APAP-induced cell death in hepatocytes^37^. As APAP-derived NAPQI causes GSH depletion in hepatocytes, it is possible that ferroptosis is involved in APAP hepatotoxicity. Indeed, Lörincz et al.^38^ previously reported that APAP-induced cell death was prevented by Fer-1 in primary mouse hepatocytes in vitro. However, the role and mechanism of ferroptosis in APAP-induced acute liver failure, especially in vivo, remained unknown^39^. In the present study, we clearly demonstrated that APAP causes hepatic lipid peroxidation and ferroptosis, resulting in acute liver failure in mice. Furthermore, APAP-induced acute liver failure is prevented by the pharmacological (i.e., Fer-1, DFO, and α-Toc) and genetic (i.e., ACSL4) inhibition of ferroptosis. To our knowledge, this study provides the first evidence that ferroptosis is the initial and predominant mechanism of cell death underlying APAP-induced liver failure in vivo.

In addition to cell death, inflammation also has a critical role in the pathogenesis of APAP-induced liver failure^24,40^. In our study, the expression of inflammatory cytokines and cell markers was not elevated at 3 h after APAP injection even though serum levels of ALT and AST reached a peak. Furthermore, we detected elevated inflammatory responses at 6 h after APAP injection (data not shown). These findings suggest that ferroptosis in hepatocytes is the initial event in the process of APAP-induced hepatotoxicity and triggers the extracellular release of cellular contents, such as DNA and high mobility group box 1, which are known as damage-associated molecular patterns (DAMPs). The released DAMPs are then recognized by Toll-like receptors or cytosolic innate immune receptors on Kupffer cells and liver endothelial cells, and eventually cause inflammatory responses. This evoked inflammatory process has recently been referred to as necroinflammation^41^. Therefore, we assume that ferroptosis is an initial event in APAP-induced acute liver failure and would be a better therapeutic target for this disorder than inflammation.

Although ferroptosis has recently been extensively studied, several issues regarding the mechanisms of lipid peroxidation or the action of Fer-1 are still controversial^23^. Some studies showed that enzymatic oxidation was induced mainly by 15-lipoxygenase (LOX) or 12-LOX during ferroptosis in certain cell lines^11^. Others showed that ferroptosis is driven by non-enzymatic peroxidation including the Fenton reaction^42^. In addition, although Fer-1 was previously considered to be a LOX inhibitor, it is now thought to be a radical trapping agent in membrane phospholipid^33^. In this regard, we and other researchers have developed new procedures to distinguish among oxidation mechanisms by analyzing the structural isomers of lipid hydroperoxides (i.e., PCOOH, fatty acid hydroperoxide, and triacylglycerol hydroperoxide) in foods as well as in serum or tissues in vivo^21,43–46^. In the present study, we showed that the isomers characteristic of auto-oxidation were increased compared with those characteristic of other oxidation mechanisms. Importantly, these findings demonstrate for the first time that auto-oxidation plays a major role in the oxidation mechanisms in APAP-induced ferroptosis. We also showed that 8-iso-PGF2α was elevated in APAP-injected liver tissue, and this elevation was inhibited by Fer-1. Taken together, our data indicate that random auto-oxidation targeting n-6 PUFA mainly contributes to lipid peroxidation in APAP-induced ferroptosis. As the mechanism of lipid oxidation is still under debate, these findings provide novel insight into the molecular mechanism of ferroptosis in various diseases in vivo.

This study has several limitations. First, although we demonstrated that ferroptosis is the cell death mechanism responsible for APAP-induced hepatotoxicity, the subcellular locations, including plasma membrane, mitochondria, and endoplasmic reticulum, and lysosome, where lipid peroxidation occurs during ferroptosis remain unclear^23,47,48^. In this regard, APAP-induced mitochondrial dysfunction has been shown to contribute to the pathogenesis of APAP-induced acute liver failure^5^, suggesting that mitochondria are a candidate site for APAP-induced membrane lipid peroxidation and ferroptosis. In support of this proposal, Gao et al.^49^ recently reported that mitochondria play a pivotal role in cysteine-deprivation-induced ferroptosis. In contrast, Gaschler et al.^48^ reported that cells lacking mitochondria still underwent ferroptosis and suggested that the presence of mitochondria is not required for ferroptosis. Therefore, the role of mitochondria in APAP-induced ferroptosis in hepatocytes remains to be elucidated. Second, to explore a therapeutic effect of Fer-1, we treated Fer-1 at 1 h after APAP injection and assessed the liver injury. However, this late treatment with Fer-1 was unable to rescue APAP-induced acute liver failure (data not shown), suggesting that inhibition of ferroptosis has a protective but not therapeutic effect on APAP-induced acute liver failure in mice. Interestingly, however, it is known that a peak level of APAP-generated protein adducts in humans is detected at later time points than in mice^50^. Indeed, the antioxidant N-acetylcysteine (NAC) is effective when administered within 8 h of APAP overdose^51^; therefore, we assume that ferroptosis inhibition (e.g., by α-Toc and DFO) may become a therapeutic option for patients with APAP overdose in a clinical setting. Furthermore, we observed that NAC significantly prevented ferroptosis in certain cells in vitro (data not shown), suggesting that the therapeutic effect of NAC is mediated through, at least in part, inhibition of ferroptosis. Thus, further investigations are necessary to elucidate the precise mechanism underlying ferroptosis and APAP-induced hepatotoxicity, and to facilitate clinical applications in the future.

In conclusion, we have demonstrated that ferroptosis has a central role in APAP-induced hepatotoxicity. Lipid peroxidation, mainly derived from arachidonic acid by auto-oxidation, leads to hepatocyte ferroptosis, resulting in acute liver failure. Furthermore, α-Toc, which is widely used as a vitamin E supplement, prevented APAP-induced acute liver failure. These findings provide new insights into the mechanism underlying APAP-induced hepatotoxicity and suggest that ferroptosis may be a therapeutic target for APAP-induced acute liver failure.

## Supplementary information

Supplementary information

supplementary Figure S1

supplementary Figure S2

supplementary Figure S3

supplementary Figure S4

supplementary Figure S5

supplementary Figure S6

supplementary Tables
