# Supplementary material for: Ferroptosis driven by radical oxidation of n-6 polyunsaturated fatty acids mediates acetaminophen-induced acute liver failure
Source: Cell Death Dis. 2020 Feb 24;11(2):144. doi: 10.1038/s41419-020-2334-2 (PMC7039960; doi:10.1038/s41419-020-2334-2)
Supplement: Supplementary file 1 — Supplementary information [file 41419_2020_2334_MOESM1_ESM.docx]

**Supplementary information**

**Ferroptosis driven by radical oxidation of n-6 polyunsaturated fatty acids mediates acetaminophen-induced acute liver failure (Yamada *et al*.)**

*LC-MS analysis for lipid mediators*

Lipid mediators in liver tissue were analyzed by an LC-MS-8060 (Shimadzu, Kyoto, Japan) as described previously (1). Briefly, after the liver was rinsed with 1 mL saline via the portal vein, liver samples were isolated and immediately frozen at –80ºC until use. The tissue samples were homogenized in methanol containing 0.1% formic acid and internal standard. The separated methanol-phase was extracted by a Strata X polymer reversed-phase column (33 μm, 10 mg; Phenomenex, Torrance, CA). The cartridges were washed with 1 mL each of 0.1% formic acid, 15% ethanol, and petroleum ether, and then the lipid was eluted with 300 μL of methanol containing 0.1% formic acid. The eluent was evaporated and reconstituted in 30 μL of methanol. The extracted lipids were analyzed by an LC-MS-8060 using the Lipid Mediator version 2 software package (Shimadzu). Deuterium-labeled 6-keto prostaglandin (PG)F1α-d4, PGF2α-d4, PGE2-d4, PGD2-d4, leukotriene (LTC) B4-d4, 15(S)-HETE-d8, 12(S)-HETE-d8, 5(S)-HETE-d8, PAF C-16-d4, and oleoyl ethanolamide-d4 were purchased from Cayman Chemical (Ann Arbor, MI) and used as internal standards.

*Analysis of lipid hydroperoxide isomers*

Mechanisms of lipid peroxidation *in vivo* by APAP administration were evaluated by analyzing lipid hydroperoxide, such as phospholipid hydroperoxide (PCOOH), isomers. Briefly, serum (200 μL) was diluted with 400 μL of 0.9% KCl aqueous solution. The solution was added to 2.4 mL of chloroform–methanol (2:1, v/v) containing 0.002% butylated hydroxytoluene. The extract was partitioned into two layers by centrifugation (2,000 × *g*, 20 min, 4 °C): the chloroform layer (lower organic layer) and the methanol–water layer (upper layer). The lower chloroform layer (lipid fraction) was collected. The remaining aqueous layer was added to 1.6 mL of chloroform–methanol (10:1, v/v) and subjected to centrifugation (2,000 × g, 20 min, 4 °C). The lower organic layer was collected and combined with the previously extracted lower layer. The combined lipid fraction was evaporated under nitrogen gas. The dried serum extract was redissolved in 600 μL of chloroform–2-propanol (2:1, v/v), and 500 μL of this mixture was loaded onto a Silica Sep-Pak cartridge (Waters, Tokyo, Japan) equilibrated with chloroform-2-propanol (2:1, v/v). The cartridge was rinsed with 1.5 mL of chloroform–2-propanol (2:1, v/v) and eluted with 1.5 mL of methanol. The eluent was evaporated and the residue was dissolved in 200 μL of methanol. A final aliquot of 20 μL was subjected to LC-MS/MS analysis. PCOOH isomers were analyzed using our previous methods with modifications (2, 3). Samples were analyzed with a 4000 QTRAP mass spectrometer (SCIEX, Tokyo, Japan) equipped with a CHIRALPAK IB-3 column (2.1 mm i.d. × 150 mm, 3 μm, Daicel, Osaka, Japan) maintained at 40°C. Total flow was set as follows: 0–13.1 min, 0.30–0.23 mL/min linear; 13.2–22.0 min, 0.3 mL/min. Gradient elution was performed using a two-solvent system consisting of solvent A (water) and solvent B (methanol). The gradient program was as follows: 0–13.1 min, 80–84.6% B linear; 13.2–22.0 min, 100% B. PCOOH isomers were detected with the following multiple reaction monitoring (MRM) ion pairs: PCOOH bearing 9-10*E*,12*Z*-hydroperoxyoctadecadienoic acid (HPODE) (9-10*E*,12*Z*-HPODE PC); *m/z* 812.7 > 388.2, 9-10*E*,12*E*-HPODE PC; *m/z* 812.7 > 388.2, 10-8*E*,12*Z*-HPODE PC; *m/z* 812.7 > 684.5, 12-9*Z*,13*E*-HPODE PC; *m/z* 812.7 > 683.5, 13-9*Z*,11*E*-HPODE PC; *m/z* 812.7 > 541.5, and 13-9*E*,11*E*-HPODE PC; *m/z* 812.7 > 541.5).

**Supplementary table**

*Primers used for real-time RT-PCR*

| Gene | Forward | Reverse |
| --- | --- | --- |
| *Actb* | 5’-CACAGCTTCTTTGCAGCTCCTT-3’ | 5’-AGCGCAGCGATATCGTCAT-3’ |
| *Ccl2* | 5’-GGCTCAGCCAGATGCGTTAAC-3’ | 5’-GCCTACTCATTGGGATCATCTTG-3’ |
| *Emr1* | 5’-CCTGGACGAATCCTGTGAAG-3’ | 5’-GGTGGGACCACAGAGAGTTG-3’ |
| *Ly6g* | 5’-TGCTCTGGAGATAGAAGTTATTGTG-3’ | 5’-TTACCAGTGATCTCAGTATTGTCCA-3’ |
| *Il1b* | 5’-TGAAGTTGACGGACCCCAAA-3’ | 5’-TGATGTGCTGCTGTGAGATT-3’ |
| *Il6* | 5’-ACAACCACGGCCTTCCCTACTT-3’ | 5’-CACGATTTCCCAGAGAACATGTG-3’ |
| *Ptgs2* | 5’-GGGAGTCTGGAACATTGTGAA-3’ | 5’-GTGCACATTGTAAGTAGGTGGACT-3’ |
| *Tnfa* | 5’-CCCCAAAGGGATGAGAGTTC-3’ | 5’-GCTTGTCACTCGAATTTTGAGAA-3’ |

**Supplementary Excel file**

The data on lipid mediators analyzed in this study are provided in a Supplementary Excel file.

**Supplementary figure legends**

*Supplementary Fig. S1. Schematic diagram of px330-sgACSL4 vector*

*Supplementary Fig. S2. Dose and time effects of APAP on hepatotoxicity*

Serum samples were obtained from mice injected with vehicle or APAP (100, 150, and 200 mg/kg) for the indicated periods after injection. Mice were treated with Fer-1 (10 mg/kg) or vehicle 1 h prior to injection. Dose and time effects of APAP on serum AST and ALT were assessed (n = 4–6 for each). Data are expressed as dot plots with mean ± SEM. **p* < 0.05, ***p* < 0.01.

*Supplementary Fig. S3. TUNEL stain*

*Supplementary Fig. S4. Iron levels in the liver tissue in vehicle- and DFO-treated mice*

Liver samples were obtained from mice injected with vehicle after injection. Mice were treated with DFO (100 mg/kg/day) or vehicle for 7 consecutive days prior to APAP injection. Iron levels were measured. Data are expressed as dot plots with mean ± SEM. ***p* < 0.01.

*Supplementary Fig. S5. Relative levels of serum PCOOH isomers*

Serum samples were obtained from mice injected with vehicle or APAP (200 mg/kg) 3 h after injection. Mice were treated with Fer-1 (10 mg/kg) or vehicle 1 h prior to injection. Six isomers of PCOOH (9-10*E*,12*Z*-HPODE PC, 9-10*E*,12*E*-HPODE PC, 10-8*E*,12*Z*-HPODE PC, 12-9*Z*,13*E*-HPODE PC, 13-9*Z*,11*E*-HPODE PC, and 13-9*E*,11*E*-HPODE PC) were analyzed by using LC-MS/MS. Data are expressed as dot plots with mean ± SEM. **p* < 0.05.

**References**

1. Yamada M, Kita Y, Kohira T, Yoshida K, Hamano F, Tokuoka SM, et al. A comprehensive quantification method for eicosanoids and related compounds by using liquid chromatography/mass spectrometry with high speed continuous ionization polarity switching. J Chromatogr B Analyt Technol Biomed Life Sci. 2015;995-996:74-84.

2. Kato S, Nakagawa K, Suzuki Y, Asai A, Nagao M, Nagashima K, et al. Liquid chromatography-tandem mass spectrometry determination of human plasma 1-palmitoyl-2-hydroperoxyoctadecadienoyl-phosphatidylcholine isomers via promotion of sodium adduct formation. Analytical biochemistry. 2015;471:51-60.

3. Ito J, Nakagawa K, Kato S, Hirokawa T, Kuwahara S, Nagai T, et al. A novel chiral stationary phase HPLC-MS/MS method to discriminate between enzymatic oxidation and auto-oxidation of phosphatidylcholine. Analytical and bioanalytical chemistry. 2016;408(27):7785-93.
