# Supplementary figures and images for: Ferroptosis driven by radical oxidation of n-6 polyunsaturated fatty acids mediates acetaminophen-induced acute liver failure
Source: Cell Death Dis. 2020 Feb 24;11(2):144. doi: 10.1038/s41419-020-2334-2 (PMC7039960; doi:10.1038/s41419-020-2334-2)

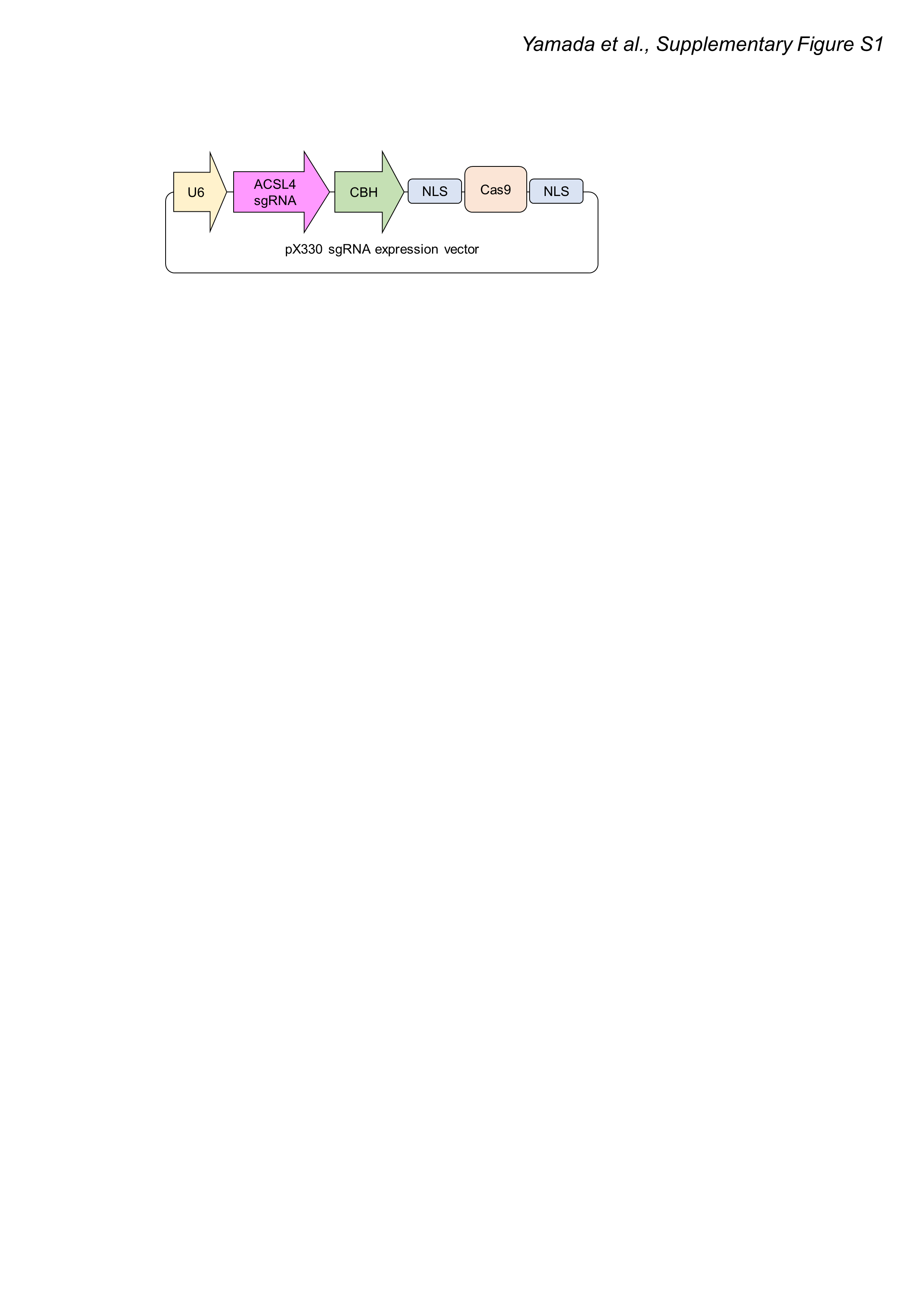

Supplement: Supplementary file 2 — supplementary Figure S1 [file 41419_2020_2334_MOESM2_ESM.tif]

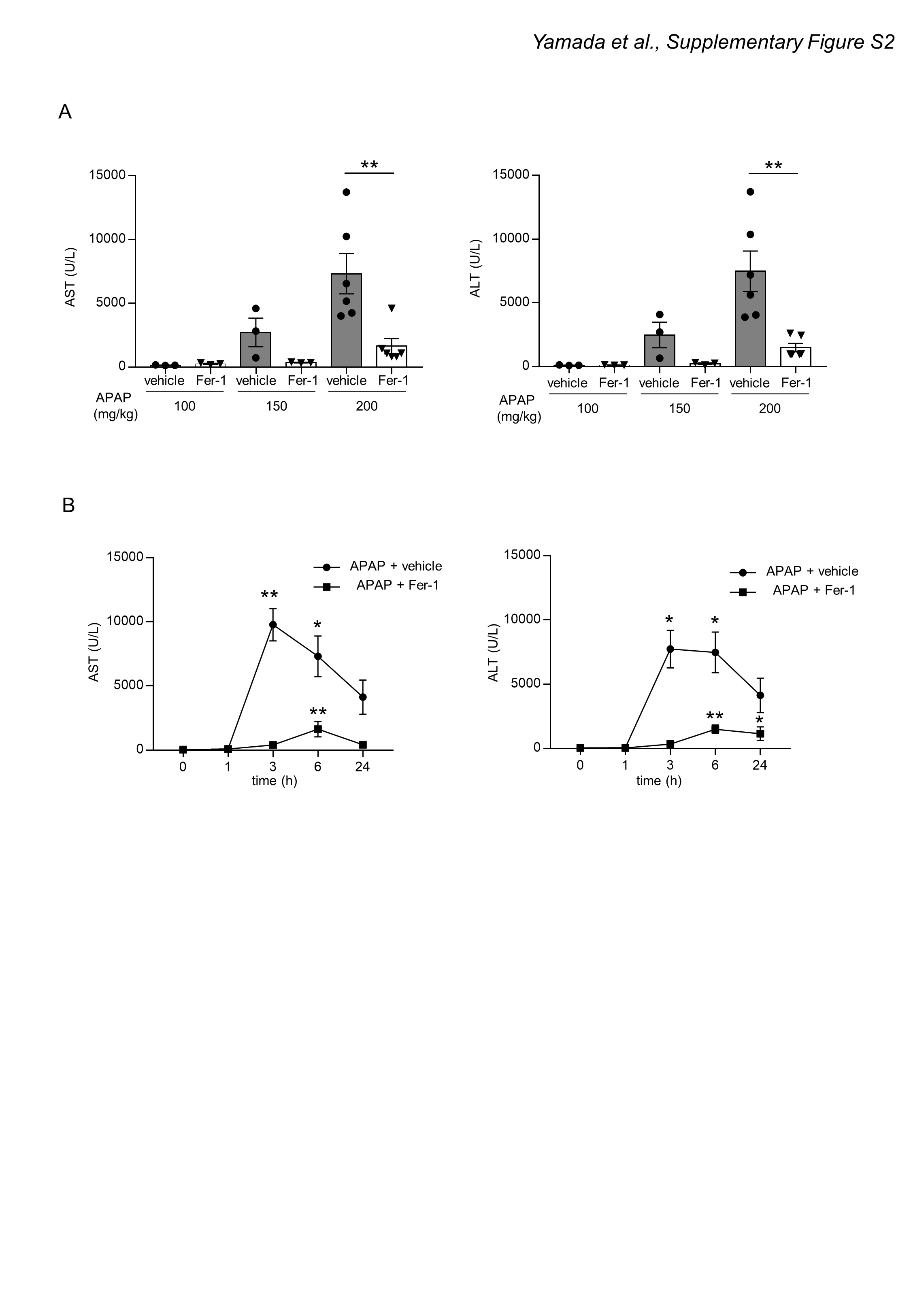

Supplement: Supplementary file 3 — supplementary Figure S2 [file 41419_2020_2334_MOESM3_ESM.tif]

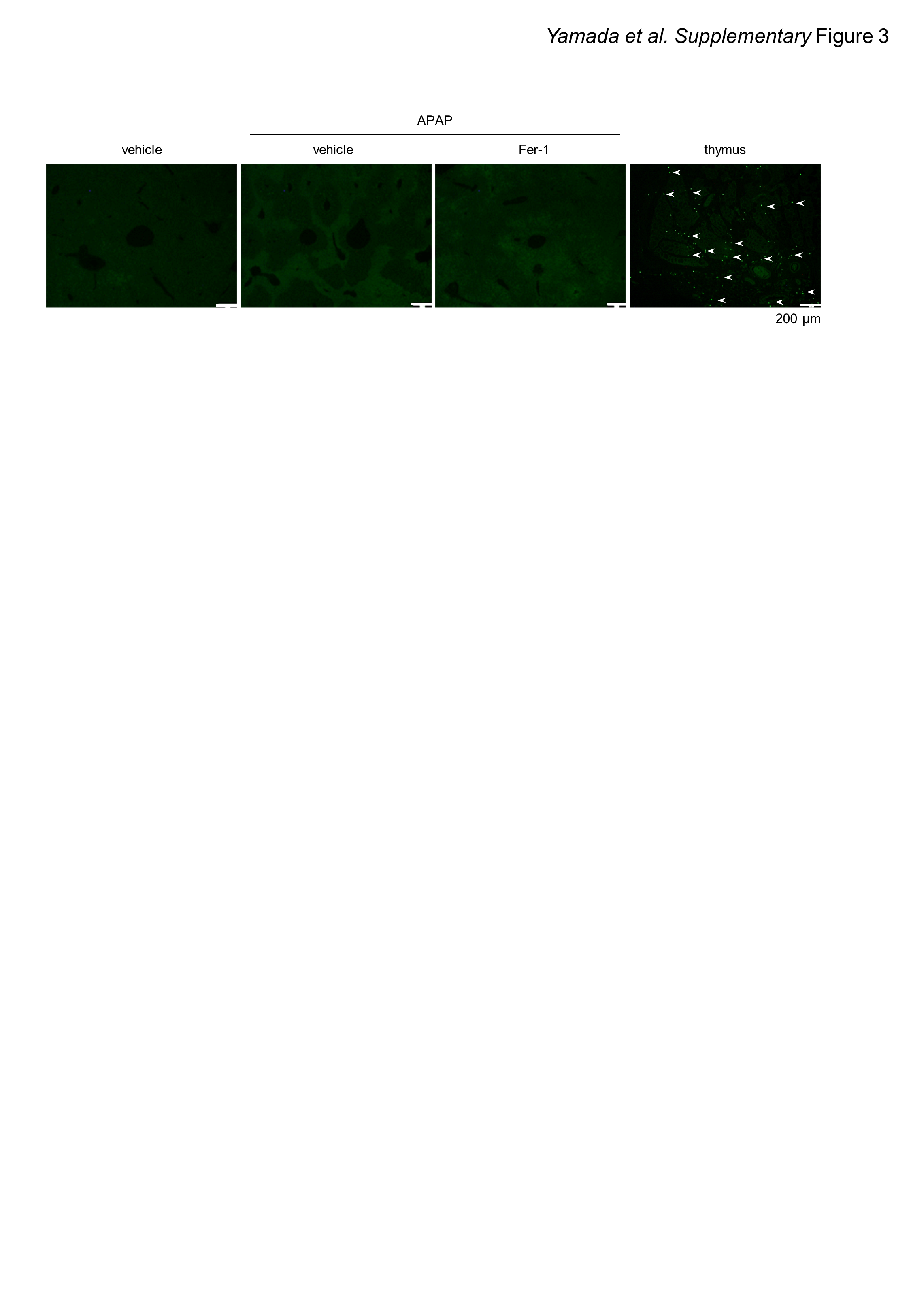

Supplement: Supplementary file 4 — supplementary Figure S3 [file 41419_2020_2334_MOESM4_ESM.tif]

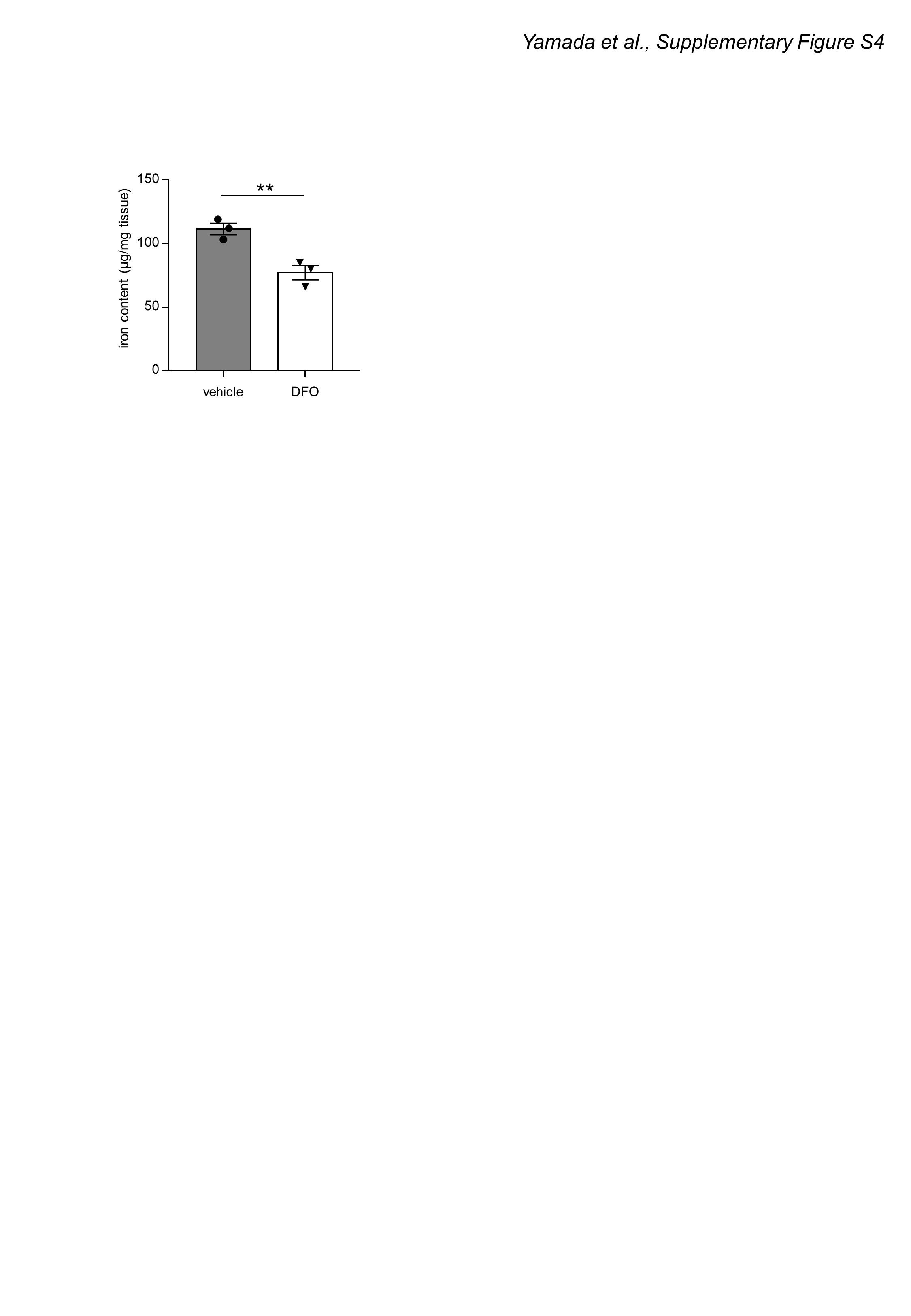

Supplement: Supplementary file 5 — supplementary Figure S4 [file 41419_2020_2334_MOESM5_ESM.tif]

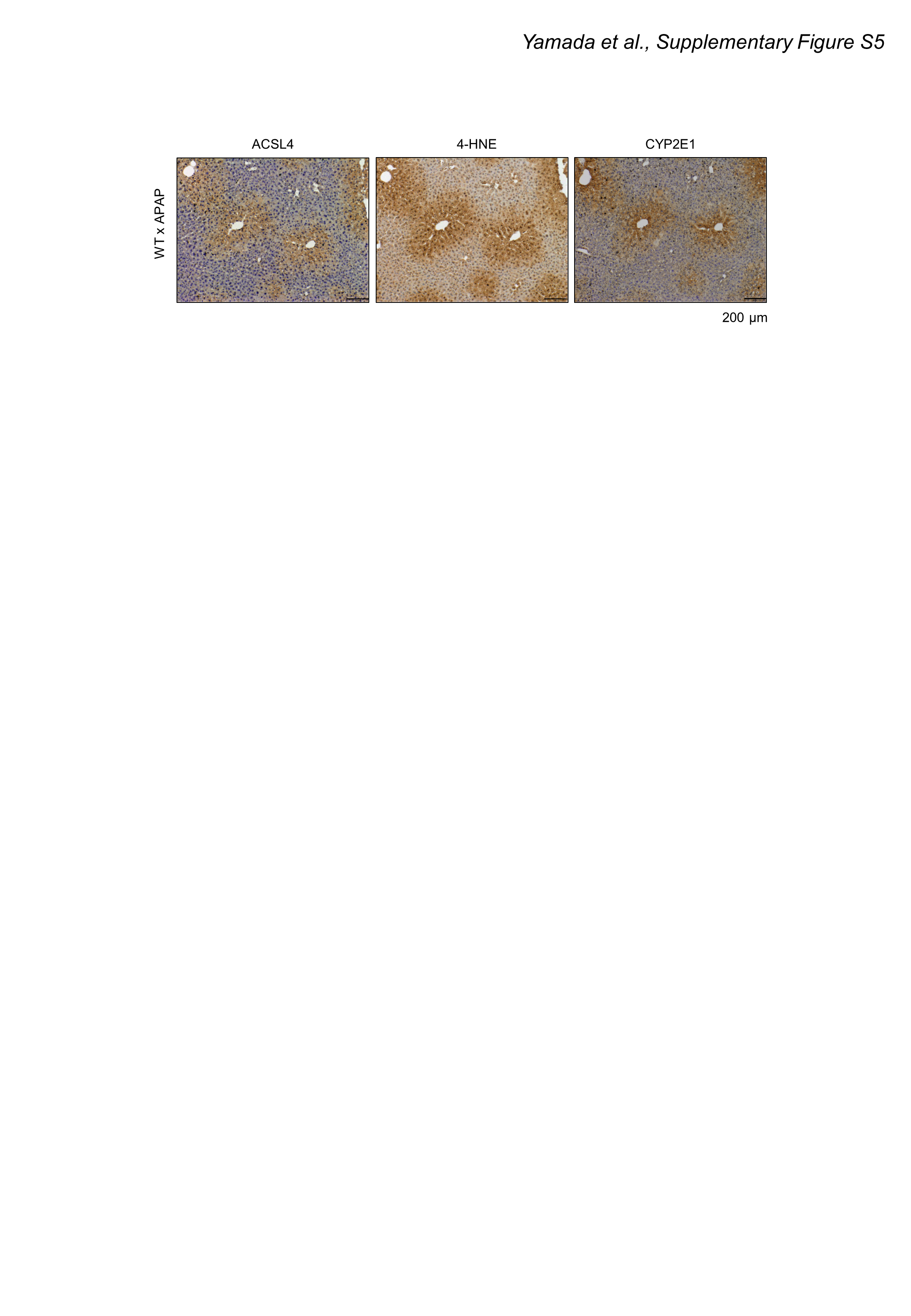

Supplement: Supplementary file 6 — supplementary Figure S5 [file 41419_2020_2334_MOESM6_ESM.tif]

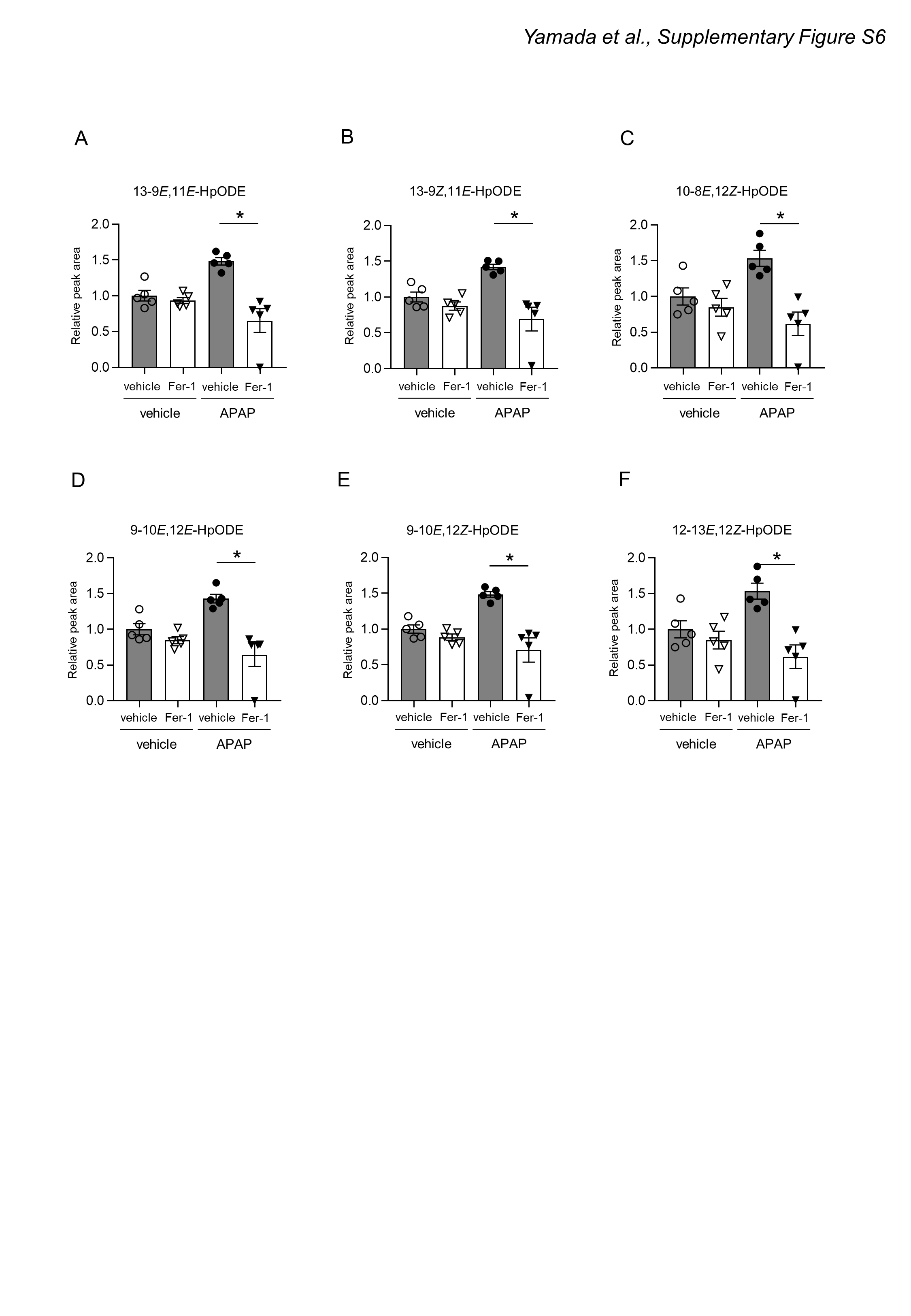

Supplement: Supplementary file 7 — supplementary Figure S6 [file 41419_2020_2334_MOESM7_ESM.tif]
